# 19.4 CAT IN FIRST-EPISODE PSYCHOSIS: FEASIBILITY, ACCEPTABILITY AND POTENTIAL TO ENHANCE VOCATIONAL RECOVERY

**DOI:** 10.1093/schbul/sby014.078

**Published:** 2018-04-01

**Authors:** Kelly Allott

**Affiliations:** Orygen, The National Centre of Excellence in Youth Mental Health

## Abstract

**Background:**

Cognitive and functioning impairments are present early in the course of psychotic disorder and remain one of the greatest treatment challenges in this population. While Cognitive Adaptation Training (CAT) is found to improve a range of outcomes in chronic schizophrenia, it has received limited investigation in first-episode psychosis (FEP). CAT may be particularly useful for addressing vocational recovery in FEP because the cognitive impairments experienced by individuals with FEP predict poorer vocational outcomes and impede the effectiveness of vocational interventions such as supported employment. The aim of this presentation is to present the findings of a pilot study investigating the feasibility and acceptability of CAT in young people with FEP and to describe the clinical considerations and adaptations required when delivering CAT with this population. Preliminary findings on the potential value of CAT in improving vocational outcomes in FEP will also be presented.

**Methods:**

This was a single-arm feasibility study of CAT conducted at the Early Psychosis Prevention and Intervention Centre (EPPIC), Melbourne, Australia. Five FEP participants received 9 months of manually-guided CAT. A range of feasibility and acceptability measures were recorded, including participant and case manager satisfaction ratings. Participants’ goals, functional needs and clinical observations and adaptations were also recorded. Formal measures of functioning, quality of life and motivation were independently administered pre- and post-intervention.

**Results:**

All participants completed the CAT intervention and session attendance rates were very high (95.3%). Participants and their case managers indicated strong satisfaction with CAT as indicated by overall positive mean ratings on the satisfaction items. CAT did not negatively affect existing case management, with case managers reporting that it enhanced their treatment. Vocational recovery (education, employment) was found to be a primary functional goal of most participants. Accordingly, the CAT intervention had a strong focus on vocational functioning, including functional domains that are requisite for successful work or educational outcomes, including organisation and planning, transportation and activities of daily living. Being mindful of factors that may be common in young FEP patients included cognitive heterogeneity, family involvement, flexibility in compensatory and environmental supports used, and the experience of stigma. There were mean improvements from baseline to post-intervention on most formal outcome measures, with the largest effects in global functioning, planning and organisation, and quality of life.

**Discussion:**

This study provides encouraging preliminary evidence that CAT is a highly feasible and acceptable intervention in FEP, which may be easily integrated within existing early intervention services. Vocational recovery is important to young people with FEP. CAT is an intervention that appears well suited to addressing this need. The effectiveness of CAT in improving functional outcomes, particularly vocational recovery in FEP warrants further investigation in a larger trial.

